# AMPKα2 knockout enhances tumour inflammation through exacerbated liver injury and energy deprivation‐associated AMPKα1 activation

**DOI:** 10.1111/jcmm.13978

**Published:** 2019-01-12

**Authors:** Shulan Qiu, Taoyan Liu, Chunmei Piao, Ying Wang, Kefang Wang, Yandong Zhou, Lun Cai, Shuai Zheng, Feng Lan, Jie Du

**Affiliations:** ^1^ Beijing Institute of Heart, Lung and Blood Vessel Diseases AnZhen Hospital Capital Medical University Beijing China; ^2^ Department of Cellular and Molecular Physiology Penn State University College of Medicine Hershey Pennsylvania; ^3^ Present address: Department of Molecular Medicine Barshop Institute for Longevity and Aging Studies University of Texas Health Science Center San Antonio Texas; ^4^ Present address: Department of Biochemistry and Molecular Biology Mayo Clinic Jacksonville Florida

**Keywords:** AMPKα1, AMPKα2 deficiency, energy deprivation, liver metastasis, macrophages

## Abstract

Tissue damage and its associated‐inflammation act as tumour initiators or propagators. AMP‐activated protein kinase (AMPK) is activated by environmental or nutritional stress factors, such as hypoxia, glucose deprivation, and other cell injury factors, to regulate cell energy balance and differentiation. We previously have reported that AMPKα2 deficiency resulted in the energy deprivation in tumour‐bearing liver and the enhanced‐hepatocyte death. In this study, AMPKα2 knockout mice and the liver metastasis model of colon cancer cells were used to address the role of AMPKα isoforms in tumour inflammation. First, we found that the AMPKα2 deficiency exacerbated the liver injury and recruitment of macrophages. Meanwhile, although compensatory expression of AMPKα1 was not significant after AMPKα2 knockout, AMPKα1 phosphorylation was elevated in remnant liver in AMPKα2 knockout mice, which was positively associated with the enhanced energy deprivation in the AMPKα2 deficient mice. Furthermore, the activated AMPKα1 in macrophage contributed to its polarizing to tumour‐associated phenotype. Thus, the enhanced tumour‐associated inflammation and activation of AMPKα1 in the AMPKα2 deficient mice may exacerbate the tumour development by affecting the tumour inflammatory microenvironment. Our study suggests that the two isoforms of AMPKα, AMPKα1 and AMPKα2 play different roles in controlling tumour development.

## Abstract 2

It is not clear whether and how the energy stress in tumour microenvironment contribute to macrophage polarization. In this study, the authors found that the AMPKα2 deficiency exacerbated the liver injury and recruitment of macrophages, and elevated AMPKα1 phosphorylation in remnant liver. The activated AMPKα1 in macrophages contributed to their polarizing to tumour‐associated phenotype. The finding provides new insights that the two isoforms of AMPKα, AMPKα1 and AMPKα2 play different roles in controlling tumour development.

## 1 INTRODUCTION

AMP‐activated protein kinase (AMPK) is a sensor and regulator activated by several factors, such as energy, nutritional, oxidative injury.^1^, ^2^, ^3^, ^4^, ^5^, ^6^ The two isoforms (α1 and α2) of AMPK catalytic subunit are enriched in specific tissues and cells. AMPKα1 is enriched in spleen and brain, but AMPKα2 is enriched in liver, skeletal muscle, kidney, and heart.^7^, ^8^, ^9^ Furthermore, AMPKα1 is localized predominantly in the cytoplasm, while AMPKα2 is localized in both the nucleus and the cytoplasm.^10^, ^11^, ^12^, ^13^ Previous studies also suggest that AMPKα1 and α2 catalytic subunits have different physiological roles, for example, AMPKα2 but not AMPKα1 affects the whole‐body energy metabolism and insulin sensitivity. However, there are limited studies focusing on whether AMPKα1 or AMPKα2 in different cells have similar or different functions during tumour development.

The stresses from a tumour, including energy/nutrient competition and inflammation, can result in the death of heathy cells. The insufficient clearance of dead cells by phagocytes can lead to pathological inflammation,^14^ which further promote the development of tumour.^15^, ^16^ Our previous study showed that hepatocyte enriches AMPKα2 and liver metastasis results in glucose deprivation and necrosis of liver hepatocytes.^17^ AMPKα2 deficiency significantly aggravated the glucose deprivation and necrosis of hepatocytes via increased ROS production. However, the role of AMPK in liver injury and inflammation remains unclear.

Although both tumour‐promoting and tumour‐antagonizing immune cells are present in the tumour microenvironment, cancer cells prefer to invoke the immune system facilitating the recruitment, activation, and persistence of the tumour‐promoting immune cells.^18^, ^19^ The polarization of macrophages is one of the prominent events after leucocyte infiltration into the tumour microenvironment. Macrophages differentiate into tumour‐associated macrophages (TAMs), ie, “M2‐like” phenotypes, in response to distinct signals such as interleukin‐4 (IL‐4), IL‐10, IL‐13 from Th2 cells, regulatory T cells (Tregs) in tumour tissues.^20^, ^21^ Abundant shreds of evidence demonstrate that TAMs are associated with poor prognosis for various types of cancer in both clinics as well as experimental animal tumour models.^22^, ^23^, ^24^ TAMs are known to facilitate the tumour metastasis, tumour‐associated angiogenesis and tumour cell invasion.^16^, ^25^ AMPK in macrophages can be activated with anti‐inflammatory cytokines, whereas pro‐inflammatory stimulus results in the inactivation of AMPK, and AMPK is suggested to promotes macrophage polarization to an M2 functional phenotype.^26^, ^27^, ^28^ Metabolic shifts, including lower glycolytic rates, increased glucose uptake and glycolysis, have been reported in anti‐inflammatory cells, such as M2 macrophages, Tregs, and quiescent memory T cells.^29^, ^30^ Altered metabolism may thus participate in the differentiation of inflammation programmes. We previously reported that AMPKα2 deficiency increased glucose deprivation in tumour‐bearing liver^17^ and glucose deprivation also existed in the large tumour masses.^31^ However, it remains unclear whether and how the energy stress in tumour microenvironment contributes to macrophage polarization.

Liver tissue is susceptible for tumour origination or metastasis and is abundant with AMPKα2. Our previous study has shown that the metastasis of colon cancer cells in liver resulted in hepatocyte necrosis through energy deprivation but not only because of the space conflict.^17^ In this study, we further exploited the role of AMPKα1/α2 isoforms in energy deprivation, liver injury, and macrophage polarization. Our results showed that AMPKα2 and AMPKα1 in distinct types of cells in the tumour microenvironment play different roles.

## 2 MATERIALS AND METHODS

### 2.1 Mouse tumour models

A liver metastasis model was carried out as described previously.^17^ SL4‐luciferase cells, a mouse colon carcinoma cell line with constitutive luciferase expression, were used for this model. SL4 cell line was transfected with a vector expressing luciferase and G418 was used for screening the stably transfected cell strain. Then the intraperitoneal injection of D‐Luciferin can show the tumour location and size. Briefly, 5 × 10^5^ SL4 cells in 100 μL of DMEM/F12 medium were intrasplenically injected into the spleen with a 26‐gauge needle after the mice were anaesthetized by intraperitoneal injection with pentobarbital (100 mg/kg). Mice were killed at the indicated day, and the blood, spleen, and liver with tumour were harvested after heart perfusion with saline. All the mice were housed in standard cages with a constant temperature range of 22 ± 2°C, 60% relative humidity and an artificial 12‐hour light/dark cycle. The study conforms to the Guide for the Care and Use of Laboratory Animals published by the U.S. National Institutes of Health (NIH publication No. 8523, revised 1996), and the protocol were approved by the Institutional Animal Care and Use Committee at Capital Medical University.

### 2.2 Tumour analysis

D‐Luciferin (15 mg/mL) was intraperitoneally injected at a dose of 10 μL/g of mouse bodyweight just before analysis. The tumour size was analysed by a Xenogen‐IVIS Lumina II device (Caliper Life Sciences, Hopkinton, MA, USA) 30 minutes after injection of 2‐Deoxyglucosone 750.

### 2.3 Alanine aminotransferase (ALT)/aspartate aminotrans‐ferase (AST) activity measurement

Fasting blood was collected from the heart of mice and then centrifuged at the speed of 1000 *g* for 5 minutes at room temperature. The activities of AST and ALT were measured by Automatic Analyzer 7020 (Hitachi, Tokyo, Japan) with the ALT, AST assay kit (BioSino Biotechnology & Science Inc., Beijing, China).

### 2.4 Flow cytometry

The composition of inflammatory cells was quantified by flow cytometry as described.^32^ Briefly, tumour tissues were cut into multiple small cubes and digested in an enzyme mixture containing collagenase type I (0.05 mg/mL) and type IV (0.05 mg/mL) and hyaluronidase (0.025 mg/mL) and DNase I (0.01 mg/mL), and soybean trypsin inhibitor (0.01 mg/mL) in DMEM for 45 minutes at 37°C. The cell suspension was centrifuged and preincubated with fragment crystallizable‐γ block antibody (anti‐mouse CD16/32; PharMingen, San Diego, CA, USA) to prevent non‐specific binding. Cell staining involved different combinations of fluorochrome‐coupled antibodies to CD45.2, F4/80, NK1.1, CD206, CD3, CD4, or CD8 (Invitrogen, Carlsbad, CA, USA) for 30 minutes at 4°C in the dark. Fluorescence data were collected using an EPICS XL Flow Cytometer (Beckman Coulter, Brea, CA, USA) and analysed by using of CellQuest (Beckman). Fluorescence minus one (FMO) controls were included to determine the level of non‐specific staining and auto‐fluorescence associated with the subset of cells in each fluorescence channel.

### 2.5 Histology and immunohistochemistry

Livers from control mice and SL4‐injected mice were harvested and then paraffin‐sectioned and stained with haematoxylin and eosin for morphological observation. For immunohistochemistry staining, the livers sections were fixed with 4% paraformaldehyde in PBS, incubated with the primary antibodies against Mac‐2 (1:200), Ki67(1:200), CD31(1:200), and TGFβ (1:200), and then incubated with the Dako ChemMateTM EnVision System (Dako, Glostrup, Denmark) for 30 minutes. Staining was visualized with use of diaminobenzidine and counterstaining with haematoxylin.

### 2.6 Western blot analysis

Protein was extracted using a Protein Extraction Kit containing protease inhibitor and protein phosphatase Inhibitor Cocktail. Equal amounts of protein extract (40 μg/lane) were separated using a 10% SDS‐PAGE gel. The blot was further probed with the primary antibodies anti‐GAPDH (1:1000), anti‐AMPKα1(1:1000), anti‐AMPKα2(1:1000), p‐AMPKα Thr172, and anti‐AMPKα (1:1000) overnight at 4°C and then fluorescent secondary antibodies (Alexa Fluor 800; Rockland Immunochemical, Gilbertsville, PA, USA) for 1 h at room temperature. Protein expression was analysed with the Odyssey infrared imaging system and Odyssey software and normalized to GAPDH expression.

### 2.7 Quantitative real‐time PCR

Total RNA was extracted with Trizol reagent (Invitrogen) according to the manufacturer's protocol and 2 μg was reversed‐transcribed using the GoScript™ Reverse Transcription system (Promega, Madison, WI, USA). For real‐time quantitative PCR, the iQ5 system (Bio‐Rad, Hercules, CA, USA) with SYBR Green I (Takara, Kusatsu, Japan) was used. Amplification was performed at 95°C for 5 minutes, 95°C for 45 seconds and 57°C for 45 seconds of each step for 45 cycles. The housekeeping gene GAPDH was used as a control. The primers used are shown below: Tumour necrosis factor‐α (TNF‐α): Forward, tcttctcattcctgcttgtgg; Reverse, ggtctgggccatagaactga; inducible nitric oxide synthase (iNOS): Forward, gggctgtcacggagatca; Reverse,ccatgatggtcacattctgc; arginase‐1 (Arg‐1):Forward, aaagctggtctgctggaaaa; Reverse, acagaccgtgggttcttcac.

### 2.8 Glucose mass spectrometry imaging

As described previously,^31^ fresh tumour‐bearing liver tissue was snap‐frozen and cryosectioned at 10 μm thickness. The slices of WT and AMPKα2 KO were transferred onto the same conductive side of indium tin oxide (ITO) slides. Desiccate the slide in vacuum for 30‐60 min. After drying, the matrix of N‐(1‐naphthyl) ethylenediamine dihydrochloride (NEDC) was applied by the Bruker ImagePrep device (Bruker Daltonics, Bremen, Germany). MALDI mass spectra were acquired at negative ion mode using a reflectron geometry MALDI‐TOF mass spectrometer (Ultraflextreme; Bruker Daltonics) equipped with a neodymium‐doped yttrium aluminum garnet (Nd:YAG)/355‐nm laser as the excitation source. Imaging data were analysed using FlexImaging v3.0 and FlexAnalysis v3.4. The intensity of glucose was shown as a false colour of the image from the mass spectrometry peak.

### 2.9 Statistical analysis

The data are expressed as means ± SD. Statistical analyses involved the use of GraphPad Prism v5.00 for Windows (GraphPad Software Inc., San Diego, CA, USA). Comparisons among more than two groups were analysed by one‐way analysis of variance followed by the Student‐Newman‐Keuls test. *P* < 0.05 was considered statistically significant.

## 3 RESULTS

### 3.1 Metastasis of colon cancer cells in liver results in greater liver injury and macrophage recruitment

**Figure 1:**
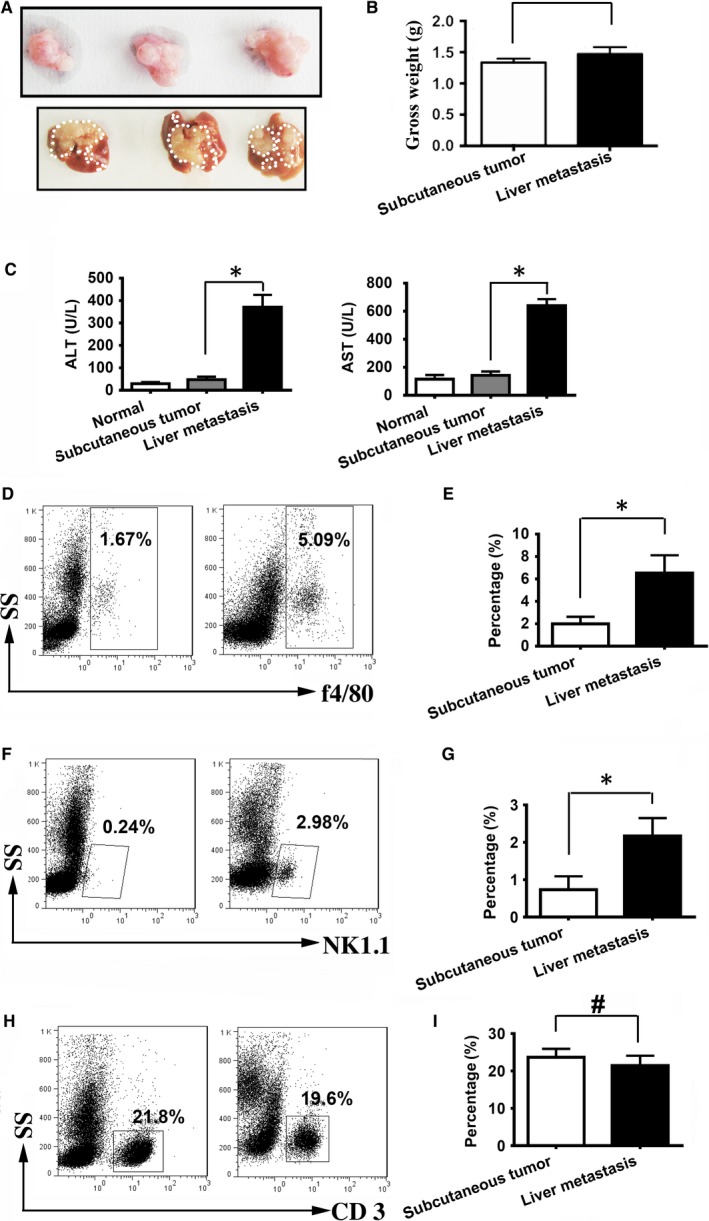
Compared to subcutaneous tumour, liver metastasis has more macrophage recruitment in blood corresponding to the liver injury. (A) Mouse colon cancer cells (SL4) were subcutaneously (top, 1 × 10^6^) or splenic (below, 5 × 10^5^) injected in C57 BL/6 WT mice. Subcutaneous tumour or tumour‐bearing livers were harvested at 14 days after injection, respectively. (B) WT subcutaneous tumour or tumour‐bearing livers were weighted, whose gross weight was similar. (C) The liver damage marker in blood, ie, ALT and AST was analysed. Liver metastasis caused much more serious liver injury. (D, E) Cytometry flow showed there was more F4/80^+^ macrophage recruitment in the blood of liver metastasis. (F, G) Cytometry flow showed there was more NK 1.1^+^ cells recruitment in the blood of liver metastasis. (H, I) Cytometry flow showed there was no difference of the CD3^+^ T cells in the blood of subcutaneous tumour and liver. n = 6, *P < 0.05; ^#^No significant difference

Cell death causes inflammation in cancer. Many previous studies showed that cancer caused liver injury.^33^, ^34^ Our previous results also suggested that colon cancer liver metastasis resulted in liver injury.^17^ To confirm whether the liver damage is associated with liver metastasis or tumour alone, we compared the liver injury in mice bearing subcutaneous tumour or mice which have liver metastasis of colon cancer cells. Figure 1A showed the gross pictures of the subcutaneous tumour and tumour‐bearing liver, and their weights were not significantly different although the liver metastasis contained the liver remnant (Figure 1B). We then examined the liver damage by measuring blood ALT and AST levels. The results showed that mice bearing liver metastasis of colon cancer cells had much higher levels of ALT and AST (subcutaneous tumour vs liver metastasis) in blood than mice with subcutaneous tumour xenograft (Figure 1C), suggesting that liver metastasis resulted in much more liver damage than the subcutaneous tumour. Since cancer increases inflammatory cells in blood,^35^ the flow‐cytometric analysis of blood cells indicated that liver metastasis caused more F4/80^+^ macrophage (Figure 1D, E) and NK1.1^+^ cells (Figure 1F, G) but not CD3^+^ T cells (Figure 1H, I) in blood, which was positively associated with the greater liver injury caused by liver metastasis.

### 3.2 AMPKα2 deficiency exacerbates liver metastasis and liver damage

**Figure 2:**
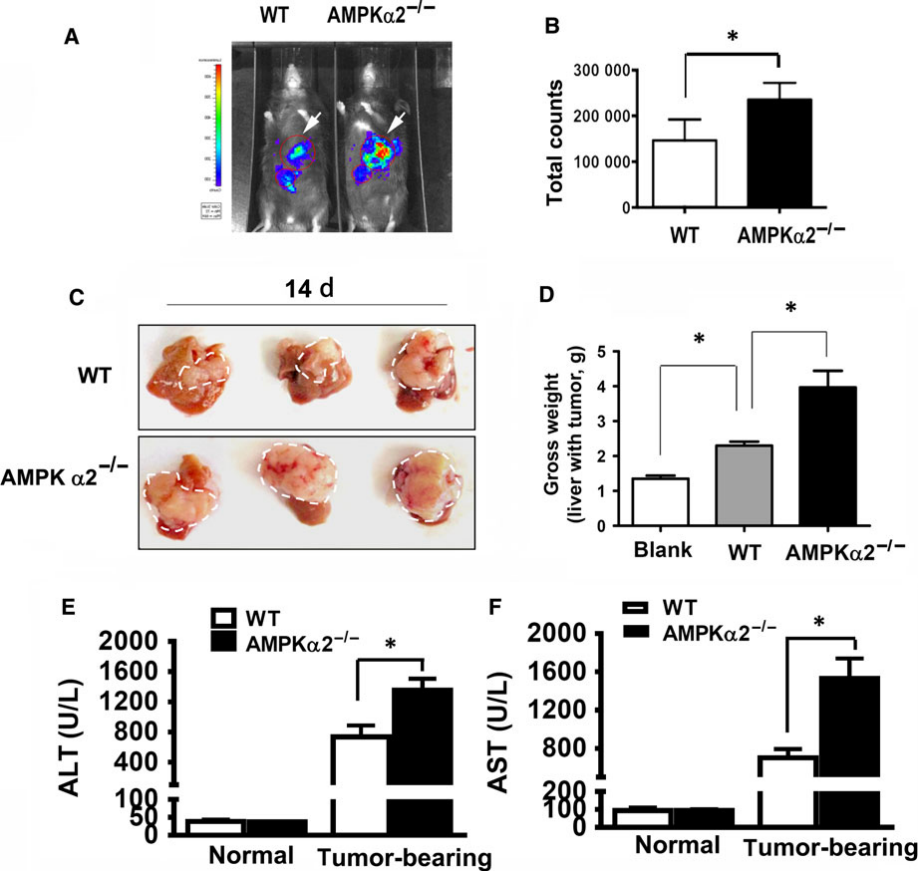
AMPK α2 deficiency enhances tumour development of colon cancer cells in liver, which was positively associated with the more serious liver injury. (A) Bioluminescence imaging after D‐luciferin injection on 12 days after SL4 injection showed the tumour location and size. (B) The analysis of total counts of bioluminescence imaging from the (A, arrowed area), n = 3. (C) Tumour‐bearing liver of 14 days after intrasplenic injection of SL4 cells (5 × 10^5^). (D) The analysis of the gross weight of tumour‐bearing liver from (C), n = 3‐5. (E) The analysis of serum ALT and AST in WT and AMPKα2^−/−^ mice corresponding to (A) (n = 5‐6). *P < 0.05; ^#^No significant difference

Previously, we reported that AMPKα2 deficiency significantly increased the liver injury and hepatocyte death.^17^ However, those results were obtained under the conditions that different numbers of SL4 cells were injected into the spleen of WT or AMPKα2^−/−^ mice, resulting similar gross weight of tumour‐bearing liver to avoid the interference of tumour size, because larger tumour size also leads to more liver damage.^17^ In this study, we injected the same number of SL4 cells into the spleen of WT or AMPKα2^−/−^ mice to investigate the roles of AMPKα2 in liver damage and tumour‐associated inflammation. First, our results showed that the deficiency of AMPKα2 increased the tumour size, which was confirmed by the bioluminescence imaging (Figure 2A, B) and the gross weight of tumour‐bearing liver (Figure 2C, D). The increased blood ALT and AST levels also indicated that AMPKα2^−/−^ mice had more severe liver damage (Figure 2E, F).

### 3.3 AMPKα2 deficiency increases the number of macrophages in blood

**Figure 3:**
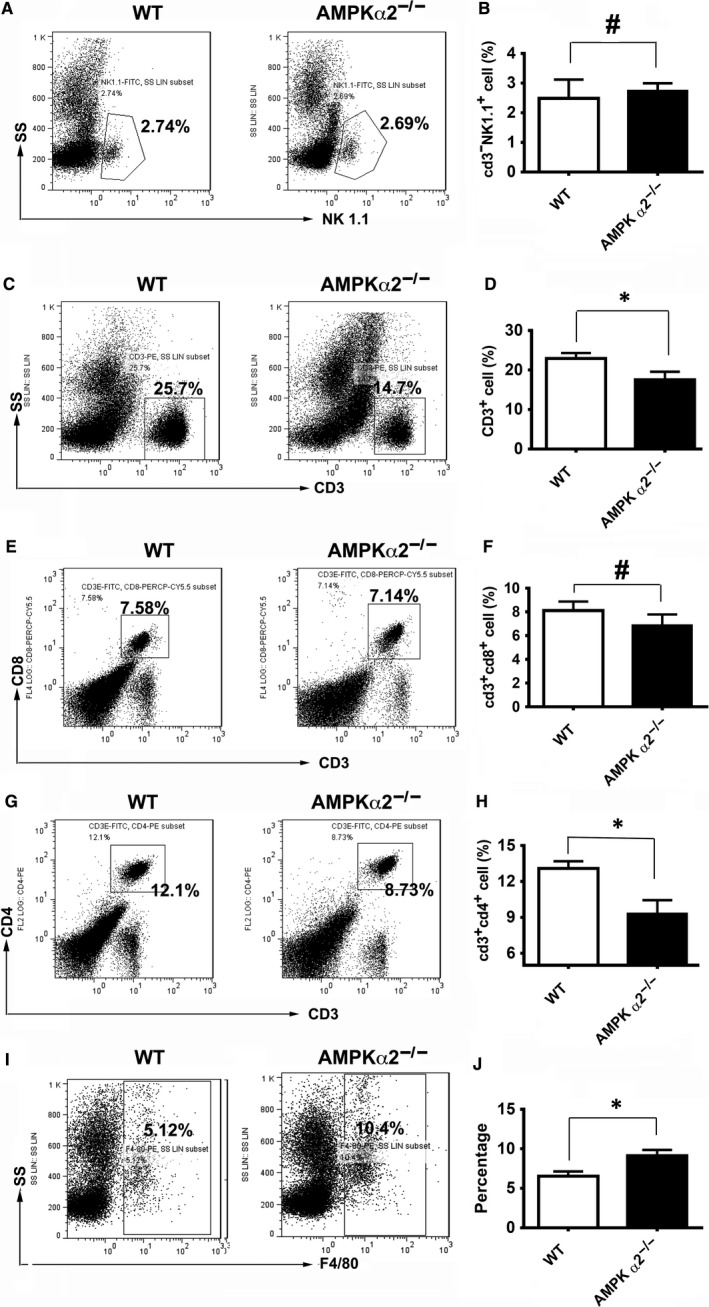
AMPKα2 deficiency exacerbates the recruitment of macrophages in blood. (A, C, E, G, I) Representative flow‐cytometry plots of NK1.1^+^ (A), CD3^+^ (C), CD3^+^ CD8^+^(E), CD3^+^ CD4^+^(G) T cells and F4/80 ^+^ (I)macrophages in blood of WT and AMPKα2^−/−^ mice at day14 after colon cancer injection; (B, D, F, H, J) Quantification of NK1.1^+^ (B), CD3^+^ (D), CD3^+^ CD8^+^(F), CD3^+^ CD4^+^(H) T cells and F4/80 ^+^ macrophages (J) in WT and AMPKα2^−/−^ mice blood. n = 3‐6, *P < 0.05; ^#^No significant difference

To investigate the association between the enhanced liver injury and tumour‐induced inflammation in the AMPKα2 deficient mice, we analysed the proportions of CD3, CD3^+^/CD4^+^, CD3^+^/CD8^+^, F4/80^+^, NK1.1^+^ cells in the blood. As shown in Figure 3A, B, NK1.1^+^ cells did not show a significant difference in the blood of WT and AMPKα2 deficient mice. However, total CD3 cells in blood decreased (Figure 3C, D), which was because of the reduction of CD3^+^/CD4^+^ cells (Figure 3G, H) but not CD3^+^/CD8^+^ cells (Figure 3E, F). And the CD3^−^/CD19^+^ cells showed no significant difference (data not shown). The decrease of blood CD4^+^ cells indicated more destruction of the immune system.^36^ Notably, there was more blood microphage in the of AMPKα2 deficient tumour‐bearing mice, which was accompanied with a more serious liver injury. Thus, our results suggest that AMPKα2 deficiency exacerbated tumour‐related inflammation.

### 3.4 AMPKα2 deficiency exacerbates infiltration of inflammatory cells in the tumour

**Figure 4:**
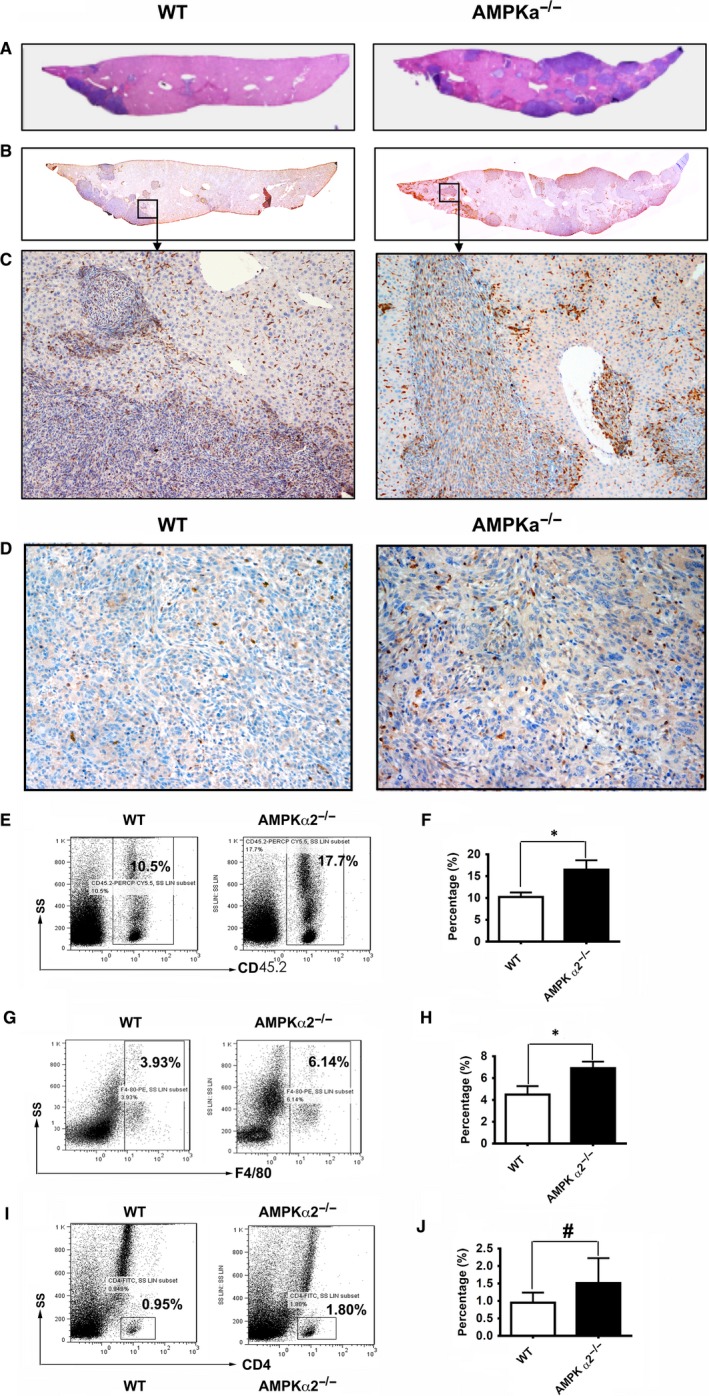
AMPKα2 deficiency exacerbates infiltration of inflammation cells in tumour. (A, B, C) Haematoxylin and eosin (HE) (A) and IHC staining with Mac‐2 antibody (B, C) at day 10; (D) IHC staining with Mac‐2 antibody at day 14; (E, G, I) Representative result of flow‐cytometry plots of CD45^+^(E), F4/80^+^ macrophages (G) and CD4^+^ T cell (I) in total cell suspensions from tumours of WT or AMPKα2^−/−^ mice at day14 after colon cancer injection; (F, H, J) Quantification and statistical analysis corresponding to (E, G, I). n = 4‐8. *P < 0.05, ^#^no significant difference

Then we analysed the immune cell infiltration, especially TAM in WT or AMPKα2^−/−^ liver‐bearing tumours. Our results of Mac‐2 immunostaining showed that there are more tumour masses scatter in livers of AMPKα2^−/−^ mice (Figure 4A). And much more Mac‐2 positive Kuffer cells infiltrated into tumour tissues of AMPKα2^−/−^ mice at day 10 (Figure 4A‐C) and day 14 (Figure 4D). We also checked the CD45^+^, F4/80^+^, CD4^+^, CD19^+^ cell populations in the tumours of WT or AMPKα2^−/−^ mice at day 14 after colon cancer cell injection. Flow cytometry result showed that CD45^+^ cell populations increased significantly in the tumours of AMPKα2^−/−^ mice (Figure 4E, F). Meanwhile, F4/80^+^ macrophages but not CD4^+^ cells increased significantly (Figure 4G‐J).

### 3.5 AMPKα2 deficiency exacerbates the differentiation of M2 macrophages

**Figure 5:**
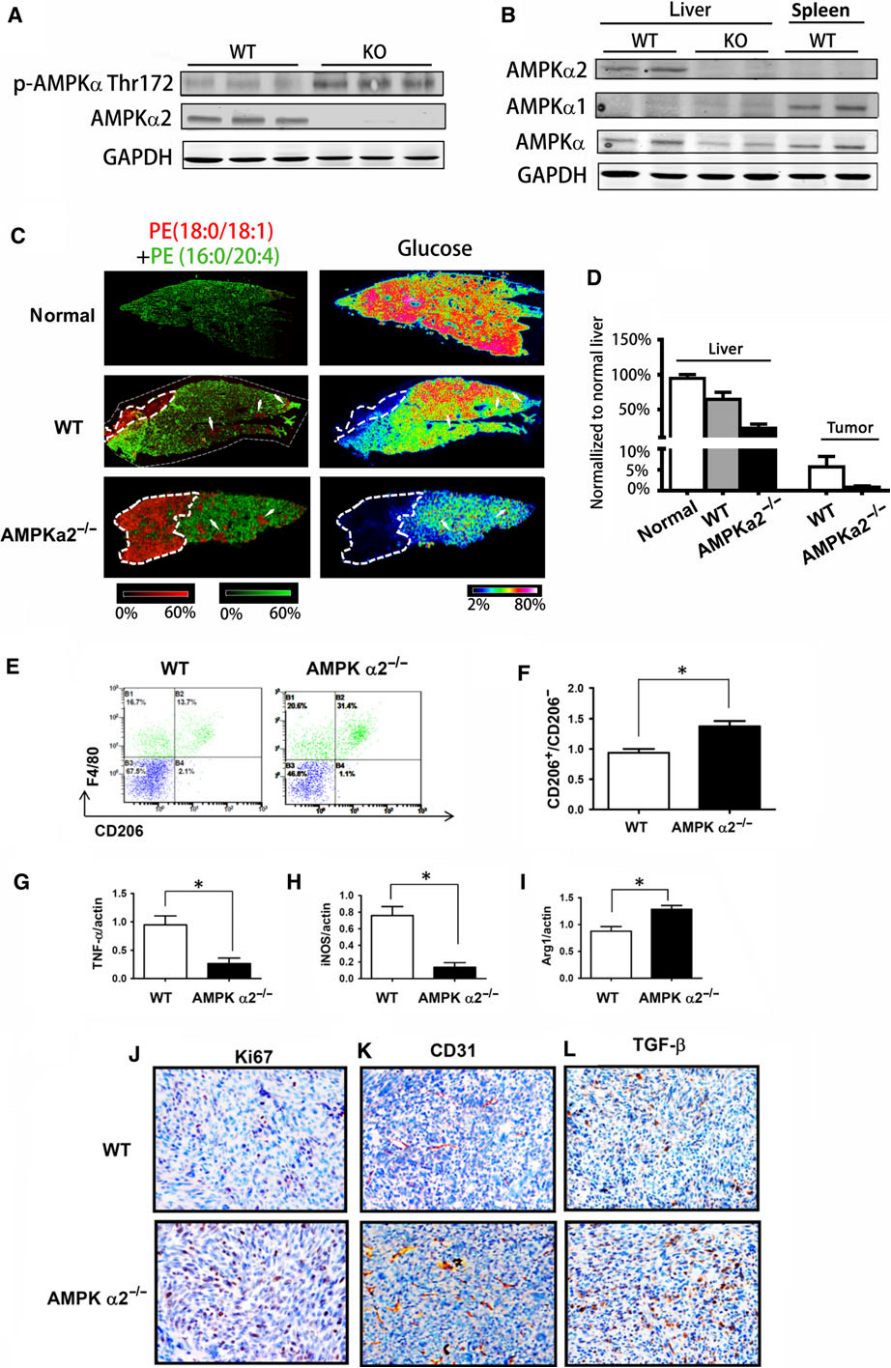
AMPKα2 deficiency exacerbates M2 type macrophages differentiation. (A) Western blot analysis for p‐AMPKα Thr172 and AMPKα2 in liver of WT and AMPKα2^−/−^ mice at day 14 after colon cancer injection; (B) Western blot analysis for AMPKα2, AMPKα1 and AMPKα expression in WT and AMPKα2^−/−^ tumour‐bearing liver, WT spleen as control; (C) Mass spectrometry imaging of glucose in normal liver and tumour‐bearing liver of WT and AMPKα2^−/−^ mice on day 14 after SL4 injection. Green is the false colour for glucose, Red is the false colour for tumour specific marker lipid as in^31^; (D) Quantification of the density of glucose in (C); (E, F) Flow‐cytometry plots and quantification of the M2 marker protein CD206 in tumour cell suspension; (G, H, I) Expression of cytokine‐related genes, TNF‐α (G), INOS (H) and Arg1(I), analysed by quantitative RT‐PCR; (J, K, L) IHC staining with Ki67(J), CD31(K) and TGFβ(L) in WT and AMPKα2^−/−^ tumour bearing liver. *P < 0.05

Our previous results showed that AMPKα2 deficiency enhanced the glucose deprivation in liver.^17^ AMPK is the sensor of energy deprivation.^37^ It was unexpected to observe that the total AMPKα phosphorylation level was higher in AMPKα2^−/−^ liver than in that of WT liver (Figure 5A), even though there was only AMPKα1 and the total AMPKα was decreased in the liver of AMPKα2^−/−^ mice (Figure 5B). And the expression of AMPKα1 did not show a difference between control and AMPKα2^−/−^ liver tissues (Figure 5B). It is well‐established that AMPKα1 but not AMPKα2 is highly expressed in macrophages, and the enhanced activation of AMPKα1 in macrophages increases the differentiation into the anti‐inflammatory M2 type,^26^, ^28^ which will promote the tumour progression.^38^ It was obvious that there were many resident Kupffer macrophage cells in the a liver and the macrophages from blood were recruited into tumour areas (Figure 4C). Thus, we used mass spectrometry imaging to check the glucose deprivation in both liver and tumour of WT and AMPKα2^−/−^ mouse. Our results showed that AMPKα2^−/−^ liver had a more serious deprivation of glucose than WT liver (Figure 5C, D). Another important result was that the glucose level in the small scattering tumour masses was similar to the surrounding liver tissue, but decreased drastically in the big tumour masses. Furthermore, both the glucose level of liver and tumours in AMPKα2^−/−^ mice were lower than those of WT mice, which is associated with the higher AMPKα phosphorylation level in the AMPKα2^−/−^ liver. We then confirmed that the big tumour masses of AMPKα2^−/−^ mice had more M2 type macrophages as shown by increased accumulation of CD206 positive macrophages (Figure 5E, F) and enhanced expression of M2 macrophages‐associated cytokines (Figure 5G‐I). Furthermore, the proliferation of tumour cells (Figure 5J), abnormal angiogenesis (Figure 5K) and TGFβ expression (Figure 5L), were all increased in the big tumour of AMPKα2^−/−^ mice, which corresponds to the anti‐inflammatory tumour environment.

## 4 DISCUSSION

The liver is the key organ controlling energy metabolism balance and acts as the most common organ for cancer metastasis. Many cancers, especially colorectal cancer, can metastasize to liver tissues, and liver metastasis is the major cause of cancer mortality.^33^, ^39^, ^40^, ^41^ Clinical investigations show that colorectal liver metastasis causes liver injury, especially at the advanced stage. Meanwhile, liver metastasis of colorectal cancer results in the increased inflammation which is related with patients’ quality of life and mortality.^42^, ^43^ Liver resection is one of the options after liver metastasis,^44^, ^45^ but there are still many patients that can not be operated, and they suffer up until late‐stage of liver metastasis. Although clinical studies report that liver metastasis is associated with liver injury,^33^, ^39^, ^46^, ^47^ limited reports have indicated the mechanism. Our previous study showed that AMPKα2 protects against liver injury from metastasized tumours via reduced glucose deprivation‐induced oxidative stress. Liver injury leads to inflammation, whereas inflammation further promotes liver injury.^14^ The inflammation acts as a propagator for tumour (reviews as 15, 48). In this study, we focused on the molecular mechanism of liver injury and inflammation. We used a subcutaneous tumour model as a control of liver metastasis to study liver injury and inflammatory cell recruitment with or without liver metastasis. Our results showed that subcutaneous tumour xenograft without liver metastasis resulted in liver injury, but liver metastasis of colon cancer cells caused much more serious liver injury, which was associated with more recruitment of F4/80^+^ macrophages but not CD3^+^ T cells. This suggests that liver metastasis exacerbates inflammatory cells recruitment and liver injury.

AMPK is a heterotrimeric serine‐threonine protein kinase, and it is conserved from yeast to mammals. The α subunit of AMPK is the catalytic subunit, which has at least two isoforms (α1 and α2) and can be phosphorylated by several upstream kinases, such as liver kinase B1 (LKB1), cAMP‐dependent kinase (PKA), and Ca^2+^/CaM‐dependent protein kinase II. AMPK is activated by multiple stress factors, including the energy stress and inflammatory environment. There are abundant reports showing that AMPK plays multiple roles in different tissues and disease conditions. The liver is enriched in AMPKα2 but not α1. Our previous report showed that liver injury was aggravated as the metastasized tumour expanded in the liver, and AMPKα2 deficiency enhanced the liver injury when the tumour sizes are similar in WT and AMPKα2^−/−^ mice (injecting fewer cancer cells into AMPKα2^−/−^ mice).^17^ Our present results showed that knockout of AMPKα2 enhanced the tumour growth and liver injury in the colon cancer cell liver metastasis mouse model (Figure 2). This suggests that the enhanced liver injury (injecting cancer cells of the same amount) in the AMPKα2 deficient mice is caused from both the bigger tumour size and the energy deprivation‐related hepatocyte death. Correspondingly, AMPKα2 deficiency exacerbated macrophage but not NK1.1^+^ nature killer T cell recruitment in the blood (Figure 3), which suggests the more serious liver injury. We also observed that the CD3^+^CD4^+^ T cells but not CD3^+^CD8^+^ T cells were decreased in the blood of the AMPKα2^−/−^ tumour‐bearing mice (Figure 3). It has been reported that cancer patients with CD4 lymphopenia have an increased risk of severe toxicity after administration of cytotoxic chemotherapy and CD4 lymphopenia can indicate the worse immune condition and identify the end‐of‐life metastatic in cancer patients.^36^ Thus, AMPKα2 deficient mice may exhibit the worse immune conditions, compared to the control mice.

Inflammation has recently been considered a hallmark of cancer and plays an important role in tumour initiation and progression.^15^, ^25^, ^49^ Tumour will recruit leucocytes and the polarization is one of the prominent events after leucocyte infiltration. In the tumour microenvironment, macrophages are one of the most abundant innate immune cells.^18^, ^21^ Meanwhile, there are substantial clinical and experimental evidence showing that these macrophages generally exhibit similarities with polarized M2 macrophages, contributing to tumour growth, invasion, and angiogenesis.^21^, ^22^ TAMs have served as a paradigm for cancer‐related inflammation.^22^, ^38^ The link between TAMs and tumour development is well‐established. Hence, we then analysed the infiltration of immune cells, including macrophages in the metastasized tumour in liver tissues. The F4/80^+^ macrophages were increased in the AMPKα2^−/−^ mice at day 14 post tumour implantations. And CD19^+^ B cells were also increased. Correspondingly, the total immune cells, ie, CD45^+^ cells, were increased. Thus, comparing the results of immune cell recruitment and infiltration from blood and tumours, the increased immune cells in the tumour was not only because of the change of blood cell but also the tumour microenvironment of liver tissues affected by AMPKα2 deficiency.

AMPK has two catalytic isoforms AMPKα1 and a2, but they are tissue‐and cell‐specific expressed. Numerous studies have shown the total effect of AMPKα1 or a2 deletion on types of mouse models, however, there is a limited study to show whether AMPKα1 and a2 play different roles in the same disease conditions although they seem to be activated by the same stimuli.^50^ Our previous results showed that AMPKα2 deficiency exacerbates the energy deprivation in AMPKα2^−/−^ tumour‐bearing liver.^17^ And we also reported that the glucose starvation in tumours is related with the tumour size and location. The glucose level was decreased significantly in the big tumour massed but not in the small tumour foci. And a glucose gradient was observed in large tumour, which implied a glucose competition between the tumour and liver remnant.^31^ Furthermore, AMPK is activated by energy stress.^37^ It was not surprising that we observed the AMPK activation was higher in the tumour‐bearing liver of AMPKα2^−/−^ mice, although the deletion of AMPKα2 resulted in decreased total AMPKα and no significant AMPKα1 compensation (Figure 5B). Thus, it suggests that AMPKα1 was more strongly activated in AMPKα2 deleted tumour‐bearing liver. It has been well‐established that AMPKα1 activation in macrophages plays an anti‐inflammatory effect.^26^, ^28^ Correspondingly, we used glucose MS‐imaging to reconfirm that the glucose level was more exacerbated in both tumour‐bearing liver and tumour tissues of AMPKα2^−/−^ mice than those of WT (Figure 5C.). It is known that the infiltrated macrophages are not only from blood but also the resident macrophages, ie, Kuffer cells (Figure 4B, C). Then we confirmed that more infiltrated macrophage was polarized into M2 type macrophages, F4/80^+^/CD206^+^ macrophages (Figure 5G, H, I).

In summary, our study showed that the two isoforms of AMPKα, AMPKα1, and AMPKα2 in different cells exploit different ways to control tumour development in the liver. Although AMPKα2 plays anti‐tumour effects, AMPKα1 in the macrophages may be activated in the tumour microenvironment and plays a pro‐tumour effect. The total AMPKα1 knockout mice or condition knockout of AMPKα1 in immune cells will help further to exploit the AMPKα1 function in tumour environment in the future studies.

## CONFLICT OF INTEREST

The authors confirm that there are no conflicts of interests.
